# Supplementary material for: Variation in life-history traits among Daphnia and its relationship to species-level responses to phosphorus limitation
Source: R Soc Open Sci. 2019 Aug 14;6(8):191024. doi: 10.1098/rsos.191024 (PMC6731724; doi:10.1098/rsos.191024)
Supplement: Supplemental material [file rsos191024supp1.docx]

Supplemental material

S1. Life-history traits for species and clones of *Daphnia.* Results of a life-history experiment that examined within and between species differences in traits. Mean trait values for seven life-history traits under two environmental conditions (high-phosphorus food – HiP; low phosphorus food –LoP) are given. Traits from left to right are as follows: core body length at the start of the experiment (mm), body length at maturation (mm), age of maturation (days), body length at the end of the experiment (mm), mean neonate body length per clutch (mm), the number of clutches, and the number of individuals in a clutch.

| Species | Start  Length (mm) | | Length  at  Maturation (mm) | | Age at  Maturation (days) | | End Length (mm) | | Mean  Clutch  Length (mm) | | Number  of  Clutches | | Mean  Clutch Size | |
| --- | --- | --- | --- | --- | --- | --- | --- | --- | --- | --- | --- | --- | --- | --- |
|  | HiP | LoP | HiP | LoP | HiP | LoP | HiP | LoP | HiP | LoP | HiP | LoP | HiP | LoP |
| ME1 | 0.56 | 0.58 | 1.25 | 1.18 | 5.71 | 7.78 | 2.33 | 1.85 | 0.50 | 0.61 | 4.90 | 4.70 | 2.65 | 1.73 |
| ME2 | 0.56 | 0.57 | 1.25 | 1.15 | 5.80 | 7.70 | 2.22 | 1.85 | 0.58 | 0.67 | 5.60 | 5.70 | 2.79 | 1.73 |
| MA1 | 0.88 | 0.88 | 3.63 | 2.97 | 8.00 | 12.50 | 3.97 | 3.65 | 0.86 | 1.02 | 4.10 | 4.20 | 5.67 | 2.45 |
| MA2 | 0.91 | 0.92 | 3.34 | 2.19 | 10.29 | 11.00 | 3.93 | 2.63 | 0.95 | 1.04 | 3.70 | 0.10 | 3.74 | 0.20 |
| MA3 | 0.78 | 0.78 | 2.91 | 2.90 | 7.89 | 15.75 | 4.25 | 2.99 | 0.87 | 0.46 | 5.60 | 0.80 | 6.15 | 0.85 |
| PX1 | 0.68 | 0.56 | 1.62 | 1.47 | 5.56 | 7.00 | 3.06 | 1.72 | 0.69 | 0.08 | 8.30 | 0.22 | 13.27 | 0.28 |
| PX2 | 0.60 | 0.60 | 1.53 | 1.33 | 5.14 | 9.56 | 2.33 | 2.07 | 0.50 | 0.58 | 5.30 | 4.27 | 5.32 | 2.21 |
| PX3 | 0.60 | 0.60 | 1.65 | 1.45 | 6.50 | 8.00 | 2.82 | 2.45 | 0.76 | 0.70 | 7.30 | 6.90 | 10.43 | 10.43 |
| OB1 | 0.58 | 0.58 | 1.51 | 1.25 | 6.00 | 7.80 | 2.98 | 2.13 | 0.68 | 0.56 | 8.60 | 1.70 | 9.02 | 2.66 |
| OB2 | 0.52 | 0.52 | 1.40 | 1.27 | 6.30 | 8.33 | 2.67 | 2.12 | 0.64 | 0.55 | 9.40 | 5.50 | 11.00 | 2.70 |
| OB3 | 0.60 | 0.61 | 1.52 | 1.35 | 6.00 | 8.11 | 2.89 | 2.08 | 0.67 | 0.42 | 9.30 | 4.30 | 13.81 | 3.44 |

S2. Principal component (PC) analysis of seven life-history traits. A Principal Component Analysis (PCA) was run for descriptive purposes in order to map out intra- and interspecific differences in multivariate space (Figure 1). This described which taxonomic (inter- vs intraspecific) level showed significant variation in life-history traits. Data collected for both treatments were run together to obtain principal components (PC), separate graphs were made for treatments for ease of viewing. Graphs were separated into a) high-P and b) low-P environments to distinguish environmental effects, with the first two principal components plotted. See Table 1 for species (letter) and clonal (number) abbreviations. Life-history traits were clustered into the two groups outlined by the PC axes, size and reproduction. PC1 loadings correlated strongly (>0.8) with size variables (start length, size at maturation, end length, and mean clutch length). PC2 loadings correlated (>0.8) with reproductive variables (clutch size, number of clutches). At the interspecific level, *D. mendotae* showed no separation from the *D. pulex/D. obtusa* group along the growth axis (PC1), indicating that these three species were of similar size, while *D. magna* (as expected) was larger (Figure 1). Along the reproductive axis (PC2), the *D. pulex/D. obtusa* group showed some separation from *D. mendotae* and *D. magna*. These results indicated that *D. pulex/D. obtusa* expressed higher fecundities. While interspecific differences were apparent (Figure 1), it was clear that intraspecific (clonal) differences also were informative. Note that *D. magna* had a broad spread of traits, while the *D. pulex/D. obtusa* group showed overlap. Shifting from high to low phosphorus, the variation along the reproductive axis (PC2) was lost, however the size axis (PC1) still exhibited variation (Figure 1). This may have indicated a shift in resource allocation from reproduction to maintenance. In addition, the separation along the growth axis was maintained between low and high P.


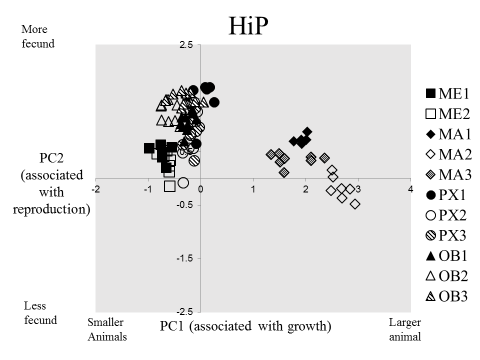


A


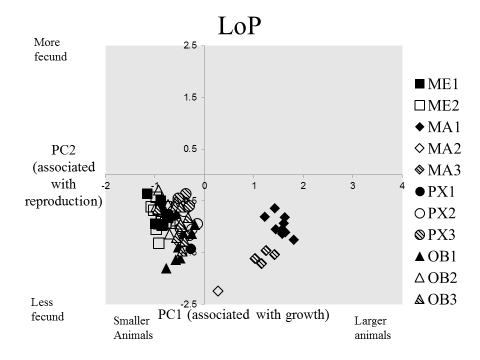


B

| **Rotated Component Matrix^a^** | | | | |
| --- | --- | --- | --- | --- |
|  | Component | | | |
|  | 1 | 2 | 3 | 4 |
| Time block | .608 | -.057 | -.180 | .259 |
| species | -.206 | .232 | .729 | .435 |
| clone | -.013 | .046 | -.060 | .906 |
| Food treatment | -.237 | -.882 | .067 | .043 |
| Maternal line | -.115 | -.063 | .828 | -.303 |
| Start length | .892 | -.135 | -.133 | -.036 |
| Length a tmaturation | .970 | .004 | -.030 | -.031 |
| End length | .902 | .360 | .054 | -.029 |
| Mean clutch length | .867 | .012 | -.154 | -.160 |
| Number of clutches | -.202 | .865 | -.030 | .014 |
| Mean clutch size | .035 | .889 | .227 | .148 |
| Extraction Method: Principal Component Analysis.   Rotation Method: Varimax with Kaiser Normalization. | | | | |
| a. Rotation converged in 5 iterations. | | | | |

S3. Effect sizes were calculated using Cohen’s d metric. Cohen’s d is a common metric of effect size, in which the means from two groups (in this case, high-P and low-P food treatments) are compared. A standard method was used instead of mean differences, so scale/unit-independent comparisons could be made between variables. The range of Cohen’s d is infinite, so comparing absolute differences between studies can be challenging, without correction. However, since all of the animals were run simultaneously in the experiment, relative differences in Cohen’s d are an appropriate metric for comparative purposes. A two-way ANOVA was run on these effects with trait type (growth or reproductive) and species as explanatory variables, and d as the response variable (species, df = 3, F = 1.64, p = 0.289; food treatment, df = 1, F = 0.009, p = 0.925; interaction, df = 3, F = 0.376, p = 0.772). Start length, length at maturation, end length were classified as growth traits. Mean clutch length, mean clutch size, and number of clutches were classified as reproductive traits.

|  | Start Length | Length at Maturation | End Length | Mean Clutch Length | Number of Clutches | Mean Clutch Size |
| --- | --- | --- | --- | --- | --- | --- |
| *D. mendotae* (2.80) | -3.13 | 1.63 | 4.59 | 1.71 | 1.23 | 4.48 |
| *D. magna* (7.01) | -0.76 | 12.42 | 12.72 | 1.11 | 4.25 | 10.82 |
| *D. pulex* (5.65) | 0.24 | 1.78 | 8.62 | -0.16 | 2.71 | 18.64 |
| *D. obtusa* (3.28) | -0.02 | 1.52 | 4.10 | 0.97 | 3.84 | 9.21 |
| **Mean effect** | **0.87** | **4.60** | **7.77** | **1.36** | **3.17** | **11.36** |
